# Real-world effects of once-daily inhaled steroid (fluticasone furoate) combined with long-acting beta-2 agonist (vilanterol) and long-acting muscarinic antagonist (umeclidinium) on lung function tests of asthma patients in Japan

**DOI:** 10.3389/fphys.2023.1131949

**Published:** 2023-04-21

**Authors:** Akira Umeda, Hisato Shimada, Tateki Yamane, Taichi Mochizuki, Yasushi Inoue, Kenji Tsushima, Kazuya Miyagawa, Atsumi Mochida, Hiroshi Takeda, Yasumasa Okada, Katsunori Masaki, Masako Matsusaka, Koichi Fukunaga

**Affiliations:** ^1^ Department of General Medicine, School of Medicine, International University of Health and Welfare (IUHW), IUHW Shioya Hospital, Yaita, Japan; ^2^ Department of Respiratory Medicine, IUHW Shioya Hospital, Yaita, Japan; ^3^ Department of Digestive Organ, IUHW Shioya Hospital, Yaita, Japan; ^4^ Respiratory Diseases Center, IUHW Mita Hospital, Tokyo, Japan; ^5^ Department of Pulmonary Medicine, School of Medicine, International University of Health and Welfare, Narita, Japan; ^6^ Department of Pharmacology, School of Pharmacy, International University of Health and Welfare, Otawara, Japan; ^7^ Department of Pharmacology, School of Pharmacy at Fukuoka, International University of Health and Welfare, Fukuoka, Japan; ^8^ Department of Internal Medicine, National Hospital Organization Murayama Medical Center, Musashimurayama, Japan; ^9^ Division of Pulmonary Medicine, Department of Medicine, Keio University, Tokyo, Japan

**Keywords:** asthma, fluticasone furoate, vilanterol, umeclidinium, small airways, peripheral airways

## Abstract

**Background:** The Japanese drug use system allowed the once-daily use of inhaled corticosteroid fluticasone furoate (FF) combined with a long-acting beta-2 agonist vilanterol (VI) and a long-acting muscarinic antagonist umeclidinium (UMEC) against asthma on 18 February 2021. We investigated the real-world effects of these drugs (FF/UMEC/VI) mainly on lung function tests.

**Methods:** This was an open-label, uncontrolled, within-group time-series (before-after) study. Prior asthma treatment (inhaled corticosteroid with/without a long-acting beta-2 agonist with/without a long-acting muscarinic antagonist) was switched to FF/UMEC/VI 200/62.5/25 μg. Subjects were evaluated by lung function tests prior to, and 1–2 months after, initiation of FF/UMEC/VI 200/62.5/25 μg. Patients were asked questions regarding the asthma control test and preference for drugs.

**Results:** Overall, 114 asthma outpatients (97% Japanese) were enrolled from February 2021 to April 2022: 104 subjects completed the study. Forced expiratory volume in 1 s, peak flow, and asthma control test score of FF/UMEC/VI 200/62.5/25 μg-treated subjects were significantly increased (*p* < 0.001, *p* < 0.001, and *p* < 0.01, respectively). In contrast with FF/VI 200/25 μg, instantaneous flow at 25% of the forced vital capacity and expiratory reserve volume were significantly increased by FF/UMEC/VI 200/62.5/25 μg (*p* < 0.01, *p* < 0.05, respectively). Sixty-six percent of subjects declared they wanted to continue FF/UMEC/VI 200/62.5/25 μg in the future. Adverse effects, mainly local, were seen in 30% of patients, but no serious adverse effects were seen.

**Conclusion:** Once-daily FF/UMEC/VI 200/62.5/25 μg was effective against asthma without serious adverse events. This is the first report that demonstrated FF/UMEC/VI dilated peripheral airways using lung function tests. This evidence on drug effects may improve our understanding of pulmonary physiology and the pathophysiology of asthma.

## 1 Introduction

Asthma is defined by a history of respiratory symptoms including wheezing, shortness of breath, dyspnea, and cough that vary over time and intensity, together with variable expiratory airflow limitation (Global Initiative for Asthma, 2022; World Health Organization) (Global Initiative for Asthma, 2022). This condition is caused by chronic airway inflammation, and the daily use of inhaled corticosteroids (ICS) is recommended for the treatment of patients with persistent asthma (Global Initiative for Asthma, 2022). The use of beclomethasone, the first ICS, was approved in 1978 in Japan. This first-generation ICS needed to be used four times a day. The Japanese drug system then approved fluticasone propionate (FP) in 1998 and budesonide in 2002, both of which needed to be used twice daily.

The combination of a long-acting β_2_-agonist (LABA) and ICS was recommended for patients at high risk of asthma exacerbations (Global Initiative for Asthma, 2022), and the first combined ICS/LABA, fluticasone propionate/salmeterol xinafoate (FP/S), was approved for use in Japan in 2007. In 2013, the first “once-daily use” of combined ICS/LABA, fluticasone furoate (FF) and vilanterol (VI), was allowed and we demonstrated the real-world efficacy and safety of this FF/VI 200/25 μg in Japanese patients with asthma (Umeda et al., 2019; Allen et al., 2013; Slack et al., 2013). We concluded that FF/VI appeared to be effective on larger airways and yielded a greater satisfaction despite a higher incidence of local effects compared to previous ICS/LABA.

Complications of asthma include asthma-chronic obstructive pulmonary disease (COPD) overlap, termed ACO (Global Initiative for Asthma, 2022). The Japanese drug use system approved the use of a triple combined drug consisting of ICS/LABA (FF/VI 100/25 μg) and a long-acting muscarinic antagonist (LAMA), umeclidinium (UMEC 62.5 μg) for COPD in May 2019. UMEC is a quinuclidine derivative and potent anticholinergic with slow functional reversibility at the human M_3_ receptor (Salmon et al., 2013; Naline et al., 2018; Lee et al., 2015). UMEC was approved as a maintenance treatment for COPD in the US, EU and Japan earlier than for asthma (Lee et al., 2015; Feldman et al., 2016; Pleasants et al., 2016; Donohue et al., 2012; 2013; Zhong et al., 2020; Singh et al., 2018; Maltais et al., 2014; Decramer et al., 2013; Tal-Singer et al., 2013; Trivedi et al., 2014). Historically, COPD was defined as a different disease compared with asthma. Currently, it is understood that COPD can be diagnosed after ruling out asthma. Therefore, it is confusing and difficult to differentiate completely between asthma and COPD (Global Initiative for Asthma, 2022).

The Japanese drug use system first approved the use of the triple combined drug consisting of ICS (FF 200 μg)/LABA (VI 25 μg)/LAMA (UMEC 62.5 μg) against asthma on 18 February 2021. Although there was a landmark report of the phase 3A random controlled trial (RCT) (CAPTAIN Study) on the data of forced expiratory volume in 1 s (FEV_1_), the efficacy of this combined drug (FF/UMEC/VI 200/62.5/25 μg) on other parameters of lung function tests has not been well known (Lee et al., 2021). The aim of this study was to evaluate the real-world efficacy of FF/UMEC/VI 200/62.5/25 μg mainly on the lung function tests for Japanese patients with asthma. Short-acting muscarinic antagonists are thought to provide less bronchodilation than short-acting β_2_-agonists in asthma patients (Ferrando et al., 2017). The factors reported to respond to anticholinergic agents include older asthmatic patients, or nocturnal or intrinsic (non-allergic) asthma (Ferrando et al., 2017; Restrepo et al., 2007). Because we wanted all patients with asthma to experience FF/UMEC/VI 200/62.5/25 μg, we performed an uncontrolled “real-world” study. After the use of FF/UMEC/VI 200/62.5/25 μg, subjects were asked which drug they preferred. This was an observational study under the control of the Japanese drug use system since 2021.

The later the expiration flow is measured, the more it reflects the resistance of very small airways (West and Luks, 2022a). We report interesting data on the instantaneous flow at 25% of the forced vital capacity (V25). This effect of additional UMEC 62.5 μg is compared with our previous study that investigated FF/VI 200/25 μg (Umeda et al., 2019). Based on the effects of these drugs, we detected important findings in the field of pulmonary physiology and the pathophysiology of asthma.

## 2 Patients and methods

This was an open-label, uncontrolled, within-group time-series (before-after) study.

### 2.1 Enrollment

We recruited asthma outpatients at the IUHW Shioya Hospital (Yaita-City, Tochigi Prefecture, Japan) from February 2021 to April 2022 (IUHW Ethics Committee according to the Declaration of Helsinki, approval number: 20-B-452). Recruitment was conducted during the outpatient service by physicians of this Hospital. All subjects provided written consent before participating in this study. Registration site and number: UMIN000047506.

Inclusion criteria included: age ≥20 years, stable asthma with the use of ICS alone or ICS/LABA or ICS/LABA/LAMA, and unstable asthma with or without the use of inhalation therapy. Patients had to be diagnosed with moderate or severe asthma by pulmonary physicians (Global Initiative for Asthma, 2018).

Exclusion criteria were pregnancy, unstable asthma that needed an increase in systemic steroids, a recent history of life-threatening asthma, and/or concomitant life-threatening disease.

### 2.2 Study procedures

Previous inhalation treatments against asthma were switched to the once-daily use of FF/UMEC/VI 200/62.5/25 μg. For asthma patients without the use of inhalation treatments, FF/UMEC/VI 200/62.5/25 μg was initiated. Subjects were evaluated by lung function tests prior to, and 1–2 months after the initiation of FF/UMEC/VI 200/62.5/25 μg. The interval was shortened from 2–3 months to 1–2 months compared with our previous study of FF/VI 200/25 μg to reduce any bias caused by “lost to follow-up” (Umeda et al., 2019). CHESTAC-8900 (CHEST M.I., INC., Tokyo, Japan) was used for the lung function tests and asthma control test (ACT) scores were recorded concurrently (Global Initiative for Asthma, 2022). At 1–2 months after the initiation of FF/UMEC/VI 200/62.5/25 μg, subjects were asked which treatment they preferred. The response to the question on patient decision making was determined by providing qualitative responses during a conversation with a physician. The response to the questions of ACT was determined by selecting responses in a questionnaire. All adverse events and respiratory symptoms were recorded.

### 2.3 Data analysis

Data are shown as the mean ± standard deviation (SD). The Student’s paired *t*-test was used for comparisons between baseline and after the use of FF/UMEC/VI 200/62.5/25 μg (two-tailed). Statistical significance was set at *p* < 0.05. For the statistical analyses, Ekuseru-Toukei 2010 (Social Survey Research Information Co., Ltd., Tokyo, Japan) was used. The sample size was estimated according to our previous study on FF/VI 200/25 μg (n = 107) (Umeda et al., 2019). To compare multiple parameters with supposedly similar power, we adjusted the sample size to approximately 107. In addition, the sample size was estimated according to our pilot study using data from the initial 30 participants. Using FEV_1_ data as the primary endpoint, the expected effect size was 0.093 L with an SD of 0.211 L; therefore, the standardized effect size was 0.44 (Browner et al., 2013). To achieve 90% power to detect significance at the level of 5% (two-sided) for a one-sample *t*-test, approximately 57 participants were required. To evaluate the other parameters, we increased the sample size to 104. We treated lung function test data that was too early for before/baseline data (more than 3 months) or too late for after data (also more than 3 months) as missing lung function test data and thus excluded these patients’ data from the analysis.

### 2.4 Additional data with the omission

In order to compare the data on FF/UMEC/VI 200/62.5/25 μg with our previous study on the switching use of FF/VI 200/25 μg (Umeda et al., 2019), we additionally calculated the data with the omission of subjects who did not use inhalation drugs for more than 3 months before the initiation of FF/UMEC/VI 200/62.5/25 μg. Although multiple regression analysis, Wilcoxon signed rank test, and Bonferroni multiple comparisons were considered, there was no appropriate method for rigorously comparing before-after studies (paired *t*-test) conducted at different time points, and it was considered appropriate to compare the results of each before-after study in parallel and observe how significant differences emerged (Armitage et al., 2002; Dawson et al., 2004; Katz, 2011).

## 3 Results

### 3.1 Study population

**FIGURE 1:**
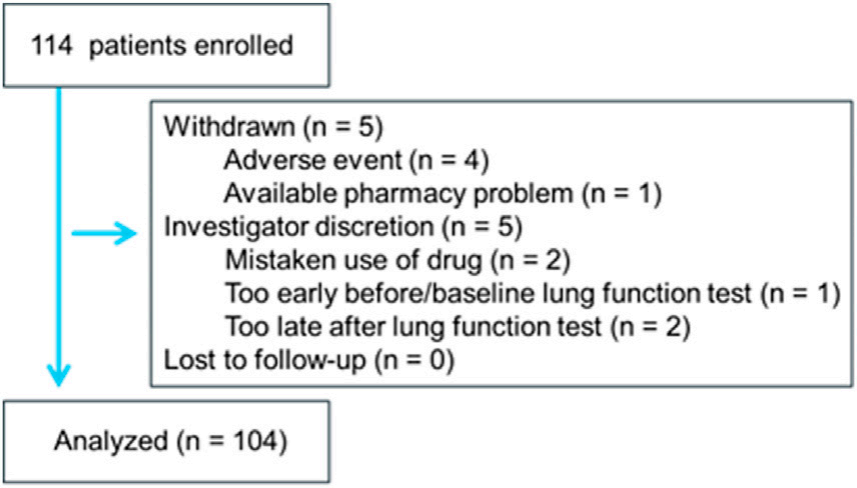
Study flow.

Overall, 114 adult patients with asthma (111 Japanese, 2 Peruvians, 1 Chinese; 57 males, 57 females; mean age: 70.2 ± 13.5 years) were enrolled from February 2021 to April 2022. Most patients were satisfied with their current treatment with regular maintenance inhalation therapy with ICS alone or ICS/LABA or ICS/LABA/LAMA. In two uncontrolled asthma patients, inhalation therapy was not used before the use of FF/UMEC/VI 200/62.5/25 μg. Patient characteristics (male/female) at baseline included: age 71.8 ± 11.8/65.4 ± 18.6 years, height 157 ± 10/159 ± 9 cm, weight 61.8 ± 14.7/61.4 ± 15.4 kg, body mass index 25.0 ± 5.1/24.0 ± 4.9 kg/m^2^; frequency of concurrent smoking 2%/2%, duration of asthma ≥5 years 68%/75%, number of exacerbations for 12 months before enrollment 0.2 ± 0.4/0.1 ± 0.3; comorbidity of emphysema 26%/21%, heart disease 18%/11%, cerebrovascular disease 12%/12%, hypertension 88%/74%, hyperlipidemia 28%/28%, and diabetes mellitus 25%/9%. The study flow is shown in Figure 1. One hundred and four subjects completed the study. Among these, 66% of subjects declared that they wanted to continue FF/UMEC/VI 200/62.5/25 μg in the future. Exacerbation of asthma with the intravenous steroid treatment was not noted in any patient during the observation period. Among 10 incomplete subjects, 4 subjects withdrew their consent because of adverse events. One subject withdrew her consent because the pharmacy which provided the brand-new drug was far from her house. One subject mistakenly used FF/UMEC/VI 200/62.5/25 μg probably because of language problems.

### 3.2 Previous drugs

**FIGURE 2:**
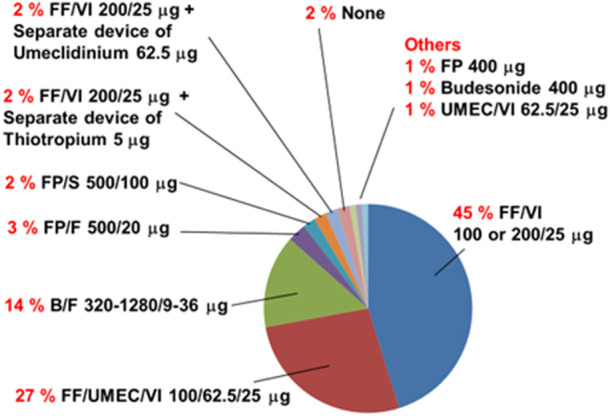
Previous inhalation drugs used before the use of the single inhaler fluticasone furoate/umeclidinium/vilanterol 200/62.5/25 μg by asthma patients (n = 104). Two percent (n = 2) of subjects with asthma had not used inhalation drugs for more than 3 months before the use of this combined drug (None). B/F, budesonide with formoterol fumarate; FF/UMEC/VI, fluticasone furoate with umeclidinium and vilanterol; FF/VI, fluticasone furoate with vilanterol; FP, fluticasone propionate; FP/F, fluticasone propionate with formoterol fumarate; FP/S, fluticasone propionate with salmeterol xinofoate; UMEC/VI, umeclidinium with vilanterol. Drug dosage values represent daily doses.

Previous inhalation drugs used before the use of FF/UMEC/VI 200/62.5/25 μg are shown in Figure 2. The most frequently used drug was FF/VI 100 or 200/25 μg. Two subjects (2%) did not use any inhalation drugs for more than 3 months before the initiation of FF/UMEC/VI 200/62.5/25 μg.

### 3.3 Change in various airflow data

Changes in various airflow data are shown in the first lines of Table 1 (n = 104). The data in the second line (bold) are the data after the omission of two subjects who did not use inhalation drugs for more than 3 months before the initiation of FF/UMEC/VI 200/62.5/25 μg (n = 102). Peak flow (*p* < 0.001), V75 (*p* < 0.001), V50 (*p* < 0.001), V25 (*p* < 0.01), V25 to height (*p* < 0.01) and maximum mid-expiratory flow rate (*p* < 0.001) were significantly increased after the (switching) use of FF/UMEC/VI 200/62.5/25 μg. The *p*-value of V50 was the smallest (0.00014) among these airflow data. The *p*-value before (1st line) and after (2nd line) the omission was consistently almost the same. Most of the statistical results (*p*-value) were similar between this study and our previous study using FF/VI 200/25 μg, except for V25, V50/V25, and V25/HT (Umeda et al., 2019).

**TABLE 1:** Changes in air flow data by the (switching) use of FF/UMEC/VI 200/62.5/25 μg and comparison between FF/UMEC/VI 200/62.5/25 μg and FF/VI 200/25 μg.

Air flow parameters	Before	After	*p*-value
Peak flow (L/s)	4.90 ± 2.08	5.16 ± 2.12	<0.001
	**4.96 ± 2.06**	**5.21 ± 2.10**	**0.001**
	5.30 ± 2.30	5.61 ± 2.31	< 0.001
V75 (L/s)	3.68 ± 2.10	4.02 ± 2.17	<0.001
	**3.72 ± 2.10**	**4.07 ± 2.17**	**< 0.001**
	3.86 ± 2.08	4.10 ± 2.18	0.010
V50 (L/s)	1.82 ± 1.15	2.00 ± 1.24	<0.001
	**1.84 ± 1.15**	**2.01 ± 1.24**	**< 0.001**
	1.88 ± 1.27	2.04 ± 1.36	< 0.001
V25 (L/s)	0.57 ± 0.40	0.63 ± 0.44	0.003
	**0.57 ± 0.40**	**0.63 ± 0.45**	**0.003**
	0.61 ± 0.53	0.64 ± 0.54	0.204
V50/V25	3.26 ± 1.04	3.26 ± 1.19	0.90
	**3.29 ± 1.04**	**3.26 ± 1.19**	**0.72**
	3.27 ± 1.07	3.40 ± 1.24	0.17
V25/HT (L/s/m)	0.36 ± 0.25	0.40 ± 0.28	0.003
	**0.36 ± 0.25**	**0.40 ± 0.28**	**0.003**
	0.38 ± 0.32	0.42 ± 0.38	0.082
MMF (L/s)	1.37 ± 0.87	1.51 ± 0.94	<0.001
	**1.34 ± 0.87**	**1.51 ± 0.94**	**< 0.001**
	1.45 ± 1.05	1.57 ± 1.09	0.005

The first line (n = 104): Comparison between before and after the (switching) use of single-inhaler fluticasone furoate 200 μg plus umeclidinium 62.5 μg plus vilanterol 25 μg (FF/UMEC/VI 200/62.5/25 μg) (paired *t*-test, two-tailed). The second line (bold, n = 102): The data of two subjects who did not use inhalation drugs for more than 3 months before the initiation of FF/UMEC/VI 200/62.5/25 μg were omitted. The third line (underlined, n = 107): The data from our previous study using FF/VI 200/25 μg (Umeda et al., 2019). Note that V25 and V25/HT were significantly increased only in the FF/UMEC/VI 200/62.5/25 μg groups. MMF, maximum mid-expiratory flow rate; V75, instantaneous flow at 75% of the forced vital capacity; V50, instantaneous flow at 50% of the forced vital capacity; V25, instantaneous flow at 25% of the forced vital capacity; V50/V25, V50 to V25; V25/HT, V25 to height.

### 3.4 Other associated parameter changes

Changes in other lung function data and associated parameters are shown in the first lines of Table 2 (n = 104). The data in the second line (bold) are the data after the omission of two subjects who did not use inhalation drugs for more than 3 months before the initiation of FF/UMEC/VI 200/62.5/25 μg (n = 102). ACT score (*p* < 0.01), FEV_1_ (*p* < 0.001), percent predicted FEV_1_ (*p* < 0.001), vital capacity (VC) (*p* < 0.05), percent predicted VC (*p* < 0.05), forced vital capacity (FVC) (*p* < 0.05), percent predicted FVC (%FVC) (*p* < 0.01), and expiratory reserve volume (ERV) (*p* < 0.05) were significantly increased after the (switching) use of FF/UMEC/VI 200/62.5/25 μg in asthma patients. The *p*-value of FEV_1_ was extremely low (0.000050). The *p*-values on VC, percent predicted VC, FVC, %FVC, and ERV after the omission were a little larger than the *p*-values before the omission. Most of the statistical results (*p*-value) were similar between this study and our previous study using FF/VI 200/25 μg, except for FVC, %FVC, ERV, and IRV (Umeda et al., 2019).

**TABLE 2:** Changes in other parameters by the (switching) use of FF/UMEC/VI 200/62.5/25 μg and comparison of the impact of rollout between FF/UMEC/VI 200/62.5/25 μg and FF/VI 200/25 μg.

Parameters	Before	After	*p*-value
ACT score	21.2 ± 4.7	22.4 ± 3.8	0.002
	**21.1 ± 4.7**	**22.5 ± 3.8**	**0.001**
	22.0 ± 3.6	23.2 ± 3.4	0.002
FEV_1_ (L)	1.78 ± 0.71	1.86 ± 0.73	<0.001
	**1.79 ± 0.71**	**1.86 ± 0.74**	**<0.001**
	1.99 ± 0.86	2.04 ± 0.86	0.006
%FEV_1_ (%)	84.0 ± 27.3	87.9 ± 28.2	<0.001
	**84.2 ± 27.0**	**87.9 ± 27.9**	**<0.001**
	84.2 ± 24.6	86.8 ± 25.9	0.008
FEV_1_ to FVC (%)	70.8 ± 13.1	71.9 ± 13.3	0.090
	**70.9 ± 13.1**	**72.0 ± 13.3**	**0.100**
	69.1 ± 13.6	70.1 ± 14.0	0.093
SpO_2_ (%)	97.0 ± 1.8	96.7 ± 1.4	0.32
	**96.9 ± 1.8**	**96.7 ± 1.4**	**0.42**
	96.4 ± 1.8	96.4 ± 1.8	0.86
VC (L)	2.64 ± 0.79	2.68 ± 0.77	0.034
	**2.64 ± 0.80**	**2.69 ± 0.78**	**0.048**
	2.90 ± 0.98	2.94 ± 0.97	0.068
%VC (%)	93.3 ± 19.6	95.2 ± 19.4	0.018
	**93.4 ± 19.6**	**95.2 ± 19.5**	**0.027**
	99.0 ± 21.0	100.5 ± 20.7	0.046
FVC (L)	2.50 ± 0.82	2.56 ± 0.82	0.014
	**2.51 ± 0.83**	**2.57 ± 0.82**	**0.024**
	2.82 ± 0.99	2.86 ± 0.97	0.14
%FVC (%)	88.2 ± 21.8	91.0 ± 21.9	0.007
	**88.4 ± 21.7**	**91.0 ± 21.8**	**0.012**
	96.2 ± 22.0	97.4 ± 22.2	0.085
ERV (L)	0.71 ± 0.41	0.76 ± 0.43	0.036
	**0.71 ± 0.41**	**0.76 ± 0.44**	**0.047**
	0.86 ± 0.52	0.86 ± 0.53	0.98
IRV (L)	1.08 ± 0.51	1.08 ± 0.48	0.83
	**1.08 ± 0.52**	**1.08 ± 0.49**	**0.97**
	1.20 ± 0.65	1.29 ± 0.62	0.011
TV (L)	0.86 ± 0.33	0.84 ± 0.33	0.56
	**0.86 ± 0.33**	**0.85 ± 0.33**	**0.69**
	0.84 ± 0.31	0.80 ± 0.28	0.12
IC (L)	1.94 ± 0.56	1.92 ± 0.56	0.54
	**1.95 ± 0.57**	**1.92 ± 0.56**	**0.54**
	2.05 ± 0.66	2.07 ± 0.69	0.60

The first line (n = 104): Comparison between before and after the (switching) use of single-inhaler fluticasone furoate 200 μg plus umeclidinium 62.5 μg plus vilanterol 25 μg (FF/UMEC/VI 200/62.5/25 μg) (paired *t*-test, two-tailed). The second line (bold, n = 102): The data of two subjects who did not use inhalation drugs for more than 3 months before the initiation of FF/UMEC/VI 200/62.5/25 μg were omitted. The third line (underlined, n = 107): The data from our previous study using FF/VI 200/25 μg (Umeda et al., 2019). Note that ERV was significantly increased only in the FF/UMEC/VI 200/62.5/25 μg groups and IRV was significantly increased only in the FF/VI 200/25 μg group. ACT, asthma control test; ERV, expiratory reserve volume; FEV_1_, forced expiratory volume in 1 s; FVC, forced vital capacity; IC, inspiratory capacity; IRV, inspiratory reserve volume; SpO_2_, oxygen saturation by pulse oximetry; TV, tidal volume; VC, vital capacity; %FEV_1_, percent predicted forced expiratory volume in 1 s; %FVC, percent predicted forced vital capacity; %VC, percent predicted vital capacity.

### 3.5 Newly seen adverse effects

**FIGURE 3:**
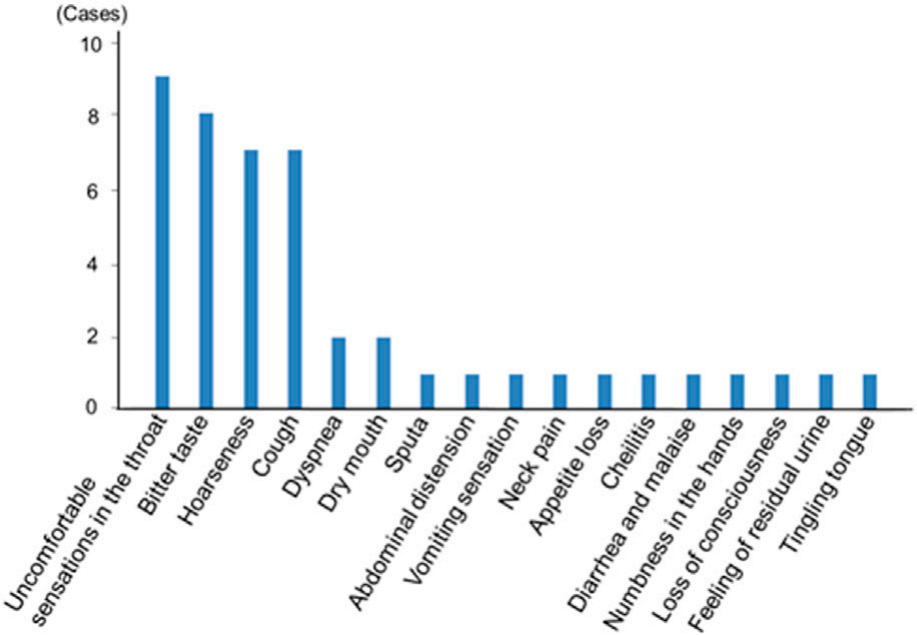
Newly seen adverse effects after the use of the single inhaler fluticasone furoate/umeclidinium/vilanterol 200/62.5/25 μg in asthma patients. Most adverse effects were related to local side effects.

Newly seen adverse effects are shown in Figure 3. Most of the adverse effects were related to local side effects including uncomfortable sensations in the throat, bitter taste, and hoarseness. There was only one case of a serious event that was loss of consciousness in a subject with an old cerebral infarction. There was no case of acute exacerbation of asthma.

### 3.6 Reasons for decision making

The main reason why subjects preferred FF/UMEC/VI 200/62.5/25 μg was a more powerful effect on asthma compared with previous drugs (Table 3). The main reason why subjects wanted to go back to previous drugs was the adverse effects of FF/UMEC/VI 200/62.5/25 μg.

**TABLE 3:** Reasons for decision making (continue or return to previous drugs).

Decision to continue FF/UMEC/VI 200/62.5/25 μg	Decision to return to previous drugs
More powerful for asthma than previous drugs (FF/VI 100/25 μg 8 cases, B/F 6 cases, FF/UMEC/VI 100/62.5/25 μg 5 cases, F/P 2 cases, FF/VI 200/25 μg plus separate device of tiotropium 1 case).Sleep better: switch from FF/VI 200/25 μg plus separate device of UMEC, 1 case).Cough decreased: switch from FF/VI 100/25 μg, B/F (2 cases, 1 case, respectively).Less effort than F/P (1 case)	Adverse effects. Discomfort sensation in the throat: switch from FF/VI 100 or 200/25 μg, B/F, FP/F, FF/UMEC/VI 100/62.5/25 μg (5 cases, 2 cases, 1 case, 1 case). Hoarseness: switch from FF/VI 100/25 μg, FP/F, FF/UMEC/VI 100/62.5/25 μg (5 cases, 1 case, 1 case). Bitter taste: switch from FF/VI 100 or 200/25 μg (6 cases). Cough: switch from FF/VI 100/25 μg, B/F, FP/F (3 cases, 2 cases, 1 case). Dry mouth: switch from FF/VI 100/25 μg, B/F (1 case, respectively). Appetite loss: switch from FF/VI 100/25 μg (1 case). Vomiting sensation: switch from FF/VI 100/25 μg (1 case). Neck pain: switch from FF/VI 200/25 μg (1 case). Numbness in hand: switch from FF/VI 100/25 μg (1 case). Diarrhea and malaise: switch from B/F (1 case). Loss of consciousness: switch from FF/UMEC/VI 100/62.5/25 μg (1 case). Feeling of residual urine: switch from FF/VI 100/25 μg (1 case). Tingling tongue: switch from FF/VI 100/25 μg (1 case). More expensive (2 cases). Less relief (1 case). Worse device than B/F (1 case)

B/F, budesonide/formoterol fumarate 320–1280/9–36 μg; FF/UMEC/VI, fluticasone furoate/umeclidinium/vilanterol; FF/VI, fluticasone furoate/vilanterol; FP/F, fluticasone propionate/formoterol fumarate 500/20 μg; FP/S, fluticasone propionate/salmeterol xinafoate 500/100 μg. Drug dosage values represent daily doses.

### 3.7 Comparison between the impact of the rollout of FF/UMEC/VI 200/62.5/25 μg and FF/VI 200/25 μg

Comparisons between the impact of the rollout of FF/UMEC/VI 200/62.5/25 μg and FF/VI 200/25 μg on various airflow data from asthma patients are shown in Table 1. The data in the third line (underlined) are from our previous study on FF/VI 200/25 μg (n = 107) (Umeda et al., 2019). Thirty-two subjects joined both studies. Peak flow, V75, V50, and maximum mid-expiratory flow rate were significantly increased by these rollouts. Nevertheless, V25 and V25 to height were significantly increased only by the rollout of FF/UMEC/VI 200/62.5/25 μg for asthma. Comparisons between the impact of the rollout of FF/UMEC/VI 200/62.5/25 μg and FF/VI 200/25 μg on other parameters are shown in Table 2. ACT score, FEV_1_, percent predicted FEV_1_, and percent predicted VC were significantly increased after the rollout of both drugs. Nevertheless, VC, FVC, %FVC, and ERV were significantly increased only by FF/UMEC/VI 200/62.5/25 μg. In contrast, inspiratory reserve volume (IRV) was significantly increased only by FF/VI 200/25 μg. These findings were consistently seen regardless of the data omission.

## 4 Discussion

By comparing the data before and after the (switching) use of FF/UMEC/VI 200/62.5/25 μg for asthma patients, we obtained the following findings. First, many lung function test data were significantly increased by the (switching) use of FF/UMEC/VI 200/62.5/25 μg. Among these data, significant increases in V25 and ERV were thought to be especially important. All data were compared with our previous study of FF/VI 200/25 μg (Umeda et al., 2019). Second, ACT scores were significantly increased by the (switching) use of FF/UMEC/VI 200/62.5/25 μg, and 66% of subjects were satisfied with this combined drug compared with previous drugs and wanted to continue using it in the future. Finally, we showed that most of the adverse events observed during FF/UMEC/VI 200/62.5/25 μg treatment were local side effects, and therefore this combined drug was thought to be safe.

The interval between the day of initiating the new inhalation drug and the day of obtaining the after data was shortened from 2–3 months to 1–2 months compared with our previous study on FF/VI 200/25 μg (Umeda et al., 2019). The number of patients “lost to follow-up” was reduced from 5/128 enrollments to 0/114 enrollments (Umeda et al., 2019). An interval of 1–2 months was selected for this before-after study of the changes in lung function to reduce the drop-out ratio. The before-after lung function data were available only from completed subjects. The after data may be worse than the before (baseline) data in patients who dropped-out of the study. If the drop-out ratio increased, the before-after data would improve more than the actual data and this would be an important limitation for the study design.

FF/UMEC/VI 200/62.5/25 μg significantly increased the Peak flow, V75, V50, and V25. In contrast, FF/VI 200/25 μg significantly increased the Peak flow, V75, and V50, but did not significantly increase V25 (Umeda et al., 2019). To the best of our knowledge, this is the first report of treatments including umeclidinium that significantly increased V25 in asthma patients. One previous study reported that tiotropium, another LAMA, significantly increased V25 in COPD patients (Yoshida et al., 2017). The later the expiration flow is measured, the more the measurement reflects the resistance of the very small airways (West and Luks, 2022a). Therefore, smaller and more peripheral bronchi seemed to be more dilated by FF/UMEC/VI 200/62.5/25 μg than FF/VI 200/25 μg.

FF/UMEC/VI 200/62.5/25 μg did not significantly increase the IRV, but significantly increased the ERV and FVC. In contrast, FF/VI 200/25 μg significantly increased IRV, but did not significantly increase ERV or FVC. These findings were consistently seen regardless of the data omission of subjects who did not use inhalation drugs for more than 3 months before the initiation of FF/UMEC/VI 200/62.5/25 μg. The main targets of FF/VI 200/25 μg seemed to be relatively larger bronchi compared with FF/UMEC/VI 200/62.5/25 μg. Adding UMEC was thought to allow the expiration of more air and increase the FVC (Papandrinopoulou et al., 2012). To the best of our knowledge, this is the first report to show UMEC in combination with ICS/LABA significantly increased the ERV in asthma patients.

LAMA drugs were first approved for COPD, then asthma (Lee et al., 2015; Feldman et al., 2016; Pleasants et al., 2016; Donohue et al., 2012; 2013; Zhong et al., 2020; Singh et al., 2018; Maltais et al., 2014; Decramer et al., 2013; Tal-Singer et al., 2013; Trivedi et al., 2014). For example, tiotropium bromide, was approved for COPD in December 2010, then it was approved for asthma in November 2014 in Japan. UMEC as an inhalation monotherapy was approved for COPD in October 2015, but to date it has not been approved for asthma in Japan. Most COPD patients have emphysema. Therefore, LAMA was thought to be mainly effective on relatively small airways that are constricted via decreased traction by the destruction of alveolar walls in emphysema (West and Luks, 2022b).

M_3_ receptors are expressed on smooth muscle cells and lung submucosal glands, and they regulate contraction, and mucus production and secretion (Ferrando et al., 2017; Cazzola et al., 2012). LAMA may mediate its effects by the bronchodilation and inhibition of mucus secretion (Ferrando et al., 2017). Furthermore, the addition of a LAMA to a LABA might strengthen the parasympathetic antagonism and stimulate sympathetic activation to achieve a greater bronchodilation effect compared with single drugs (Ferrando et al., 2017; Calzetta et al., 2015; Fukunaga et al., 2016).

Interestingly, Ikeda et al. (2012) paradoxically reported a greater distribution of M_3_ muscarinic acetylcholine receptors on larger bronchi compared with smaller bronchi in human specimens obtained by lobectomy or pneumonectomy from lung cancer patients. Their competitive binding experiments showed that the greatest distribution of M_3_ receptors was on segmental bronchi, followed by subsegmental bronchi, then in lung parenchyma (Ikeda et al., 2012). Inversely, they showed the lowest distribution of β_2_ adrenergic receptors was on the segmental bronchi, followed by the subsegmental bronchi, and then the lung parenchyma (Ikeda et al., 2012). Therefore, the effects of FF/UMEC/VI 200/62.5/25 μg on small and peripheral bronchi might be related to synergistic effects between FF, VI, and UMEC rather than the sole additional effect of UMEC (Fukunaga et al., 2016; Pera and Penn, 2014; Liu et al., 2015). Another explanation is that the ligands of agonists/antagonists to M_3_ receptors and the bronchoconstriction/dilation actions might not always be linked. UMEC ligands for M_3_ receptors may not always cause a one-to-one correlation to the effects of bronchodilation.

Most of the adverse events noted were thought to be due to the local effects of FF/UMEC/VI 200/62.5/25 μg. The only serious event (loss of consciousness) was thought to be mainly due to an old cerebral infarction. Therefore, there were no serious side effects related to this drug combination.

The main limitation of our study was the weakness of the “real-world” design. An RCT is usually more powerful than a “real-world” uncontrolled trial (Newman et al., 2013). Observed changes might be due to improved medication adherence while participating in the study, or the placebo effect of participating in a longitudinal study. Recently, the CAPTAIN Study reported the findings of a large scale RCT comparing FF/UMEC/VI (200/62.5/25 μg, n = 408) with FF/VI (200/25 μg, n = 406) against inadequately controlled asthma (Lee et al., 2021). According to that study, adding UMEC improved lung function (FEV_1_), but it did not lead to a significant reduction in moderate and/or severe exacerbations. Clinically meaningful changes, such as in acute exacerbation frequency, can be observed by longer use of drugs; therefore, RCTs with a longer observation period may be better than our before-after study. The CAPTAIN Study observed asthmatic patients for 24 weeks and reported the efficacy and safety of FF/UMEC/VI 200/62.5/25 μg. However, data related to V25, ERV, and IRV were not reported in the RCT. The strength of our study is that we observed the precise changes in lung function and that all participants received FF/UMEC/VI 200/62.5/25 μg. It was evident that the majority (66%) of subjects preferred FF/UMEC/VI 200/62.5/25 μg to previous drugs. On the other hand, 93% of subjects preferred FF/VI 200/25 μg to previous drugs in our previous study (Umeda et al., 2019). The impact of the rollout of once-daily use of ICS/LABA including FF (FF/VI 200/25 μg) may be bigger than the rollout of FF/UMEC/VI 200/62.5/25 μg for asthma patients in Japan. The increase in V25 and/or ERV in this study was not thought to link great or specific satisfaction to asthma patients. Therefore, the additional dilation of peripheral airways by FF/UMEC/VI 200/62.5/25 μg may not be clinically detectable to physicians.

While RCTs are the gold standard for establishing the safety and efficacy of new drugs, an RCT with a longer duration of drug use may yield a disadvantage for patients. For instance, even if a physician feels that arm B is clearly better than arm A in a patient assigned to the arm A group at 4 weeks, this patient cannot be shifted from arm A to arm B for 24 weeks in an RCT such as the CAPTAIN Study. This situation is not ideal for patients in the real world. In the real world, physicians and patients only seek the best treatment for the individual patient. Therefore, the free selection of treatments after a short duration (e.g., 1–2 months) of the use of a new drug is more realistic. This is an additional strength of our study.

Although multiple regression analysis, Wilcoxon signed rank test, and Bonferroni multiple comparisons were considered, there was no appropriate method for rigorously comparing before-after studies (paired *t*-test) conducted at different time points, and it was considered appropriate to compare the results of each before-after study in parallel and observe how significant differences emerged (Armitage et al., 2002; Dawson et al., 2004; Katz, 2011). This method revealed a pattern in which only FF/VI 200/25 μg significantly increased IRV, and only FF/UMEC/VI 200/62.5/25 μg significantly increased V25 and ERV. Thus, we found that FF/VI 200/25 μg mainly dilated the central airways upon rollout, while FF/UMEC/VI 200/62.5/25 μg dilated the peripheral airways upon rollout.

## 5 Conclusion

In conclusion, FF/UMEC/VI 200/62.5/25 μg was effective against asthma without any serious adverse events. Adding UMEC 62.5 μg to FF/VI 200/25 μg was thought to dilate the peripheral and very small airways additionally or synergistically. This is the first report that demonstrated FF/UMEC/VI 200/62.5/25 μg significantly increased V25 and ERV in asthma patients. Therefore, this is the first report that demonstrated FF/UMEC/VI 200/62.5/25 μg dilated peripheral and very small airways using lung function tests. This evidence on drug effects may improve our understanding of pulmonary physiology and the pathophysiology of asthma.

## Figures and Tables

**Table:** 

**ACO**	asthma-COPD overlap
**ACT**	asthma control test
**B/F**	budesonide with formoterol fumarate
**COPD**	chronic obstructive pulmonary disease
**ERV**	expiratory reserve volume
**FEV**_**1**_	forced expiratory volume in 1 s
**FF**	fluticasone furoate
**FF/UMEC/VI**	fluticasone furoate with umeclidinium and vilanterol
**FF/VI**	fluticasone furoate with vilanterol
**FP**	fluticasone propionate
**FP/F**	fluticasone propionate with formoterol fumarate
**FP/S**	fluticasone propionate with salmeterol xinafoate
**FVC**	forced vital capacity
**IC**	inspiratory capacity
**ICS**	inhaled corticosteroids
**ICS/LABA**	ICS with LAMA
**ICS/LABA/LAMA**	ICS with LABA and LAMA
**IRV**	inspiratory reserve volume
**IUHW**	International University of Health and Welfare
**LABA**	long-acting β_2_-agonist
**LAMA**	long-acting muscarinic antagonist
**MMF**	maximum mid-expiratory flow rate
**RCT**	random controlled trial
**SpO**_**2**_	oxygen saturation by pulse oximetry
**TV**	tidal volume
**UMEC**	umeclidinium
**UMEC/VI**	umeclidinium with vilanterol
**UMIN**	University Hospital Medical Information Network
**VC**	vital capacity
**VI**	vilanterol
**V25**	instantaneous flow at 25% of the forced vital capacity
**V25/HT**	V25 to height
**V50**	instantaneous flow at 50% of the forced vital capacity
**V50/V25**	V50 to V25
**V75**	instantaneous flow at 75% of the forced vital capacity
**%FEV**_**1**_	percent predicted forced expiratory volume in 1 s
**%FVC**	percent predicted forced vital capacity
**%VC**	percent predicted vital capacity

## Data Availability

The raw data supporting the conclusion of this article will be made available by the authors, without undue reservation.

## References

[B1] Allen A. Bareille P. J. Rousell V. M. (2013). Fluticasone furoate, a novel inhaled corticosteroid, demonstrates prolonged lung absorption kinetics in man compared with inhaled fluticasone propionate. Cli. Pharmacokinet. 52 (1), 37–42. 10.1007/s40262-012-0021-x PMC3693428 23184737

[B2] Armitage P. Berry G. Matthews J. N. S. (2002). Statistical methods in medical Research (4th ed.). Blackwell Publishing. Massachusetts, USA.

[B3] Browner W. S. Newman T. B. Hulley S. B. (2013). “Estimating sample size and power: Applications and examples,” in Designing clinical Research. Editors Hulley S. B. Cummings S. R. Browner W. S. Grady D. G. Newman T. B. (Philadelphia, USA: Lippincott Williams & Wilkins, a Wolters Kluwer business), 55–83.

[B4] Calzetta L. Matera M. G. Cazzola M. (2015). Pharmacological interaction between LABAs and LAMAs in the airways: Optimizing synergy. Eur. J. Pharmacol. 761, 168–173. 10.1016/j.ejphar.2015.05.020 25981302

[B5] Cazzola M. Page C. P. Calzetta L. Matera M. G. (2012). Pharmacology and therapeutics of bronchodilators. Pharmacol. Rev. 64 (3), 450–504. 10.1124/pr.111.004580 22611179

[B6] Dawson B. Trapp R. G. (2004). Basic & clinical biostatistics. New York, USA: Lange Medical Books/McGraw-Hill.

[B7] Decramer M. Maltais F. Feldman G. Brooks J. Harris S. Mehta R. (2013). Bronchodilation of umeclidinium, a new long-acting muscarinic antagonist, in COPD patients. Respir. Physiol. Neurobiol. 185 (2), 393–399. 10.1016/j.resp.2012.08.022 23026438

[B8] Donohue J. F. Anzueto A. Brooks J. Mehta R. Kalberg C. Crater G. (2012). A randomized, double-blind dose-ranging study of the novel LAMA GSK573719 in patients with COPD. Respir. Med. 106 (7), 970–979. 10.1016/j.rmed.2012.03.012 22498110

[B9] Donohue J. F. Maleki-Yazdi M. R. Kilbride S. Mehta R. Kalberg C. Church A. (2013). Efficacy and safety of once-daily umeclidinium/vilanterol 62.5/25 mcg in COPD. Respir. Med. 107, 1538–1546. 10.1016/j.rmed.2013.06.001 23830094

[B10] Feldman G. Maltais F. Khindri S. Vahdati-Bolouri M. Church A. Fahy W. A. (2016). A randomized, blinded study to evaluate the efficacy and safety of umeclidinium 62.5 μg compared with tiotropium 18 μg in patients with COPD. Int. J. Chron. Obstruct. Pulmon. Dis. 11, 719–730. 10.2147/COPD.S102494 27103795 PMC4827908

[B11] Ferrando M. Bagnasco D. Braido F. Baiardini I. Passalacqua G. Puggioni F. (2017). Umeclidinium for the treatment of uncontrolled asthma. Expert opi. Investig. Drugs 26 (6), 761–766. 10.1080/13543784.2017.1319472 28406326

[B12] Fukunaga K. Kume H. Oguma T. Shigemori W. Tohda Y. Ogawa E. (2016). Involvement of Ca2+ signaling in the synergistic effects between muscarinic receptor antagonists and β_2_-adrenoceptor agonists in airway smooth muscle. Int. J. Mol. Sci. 17 (9), 1590. 10.3390/ijms17091590 27657061 PMC5037855

[B13] Global Initiative for Asthma. Global strategy for asthma management and prevention, 2018. [accessed 2022 May]. wms-GINA-2018-report-V1.3-002.pdf (ginasthma.org), WI 53125, USA, Global Initiative for Asthma

[B14] Global Initiative for Asthma. Global strategy for asthma management and prevention, 2022. [accessed 2022 May]. https://ginasthma.org/wp-content/uploads/2022/05/GINA-Main-Report-2022-FINAL-22-05-03-WMS.pdf.

[B15] Ikeda T. Anisuzzaman A. S. Yoshiki H. Sasaki M. Koshiji T. Uwada J. (2012). Regional quantification of muscarinic acetylcholine receptors and β-adrenoceptors in human airways. Br. J. Pharmacol. 166 (6), 1804–1814. 10.1111/j.1476-5381.2012.01881.x 22300233 PMC3402805

[B16] Katz M. H. (2011). Multivariable analysis: A practical guide for clinicians and public Health researchers (3rd ed.). Cambridge University Press. University Printing House, Cambridge CB2 8BS, UK.

[B17] Lee L. A. Bailes Z. Barnes N. Boulet L. P. Edwards D. Fowler A. (2021). Efficacy and safety of once-daily single-inhaler triple therapy (FF/UMEC/VI) versus FF/VI in patients with inadequately controlled asthma (CAPTAIN): A double-blind, randomised, phase 3A trial. Lancet Respir. Med. 9 (1), 69–84. 10.1016/S2213-2600(20)30389-1 32918892

[B18] Lee L. A. Briggs A. Edwards L. D. Yang S. Pascoe S. (2015). A randomized, three-period crossover study of umeclidinium as monotherapy in adult patients with asthma. Respir. Med. 109 (1), 63–73. 10.1016/j.rmed.2014.10.009 25464907

[B19] Liu Y. H. Wu S. Z. Wang G. Huang N. W. Liu C. T. (2015). A long-acting β2-adrenergic agonist increases the expression of muscarine cholinergic subtype-3 receptors by activating the β2-adrenoceptor cyclic adenosine monophosphate signaling pathway in airway smooth muscle cells. Mol. Med. Rep. 11 (6), 4121–4128. 10.3892/mmr.2015.3307 25672589 PMC4394984

[B20] Maltais F. Singh S. Donald A. C. Crater G. Church A. Goh A. H. (2014). Effects of a combination of umeclidinium/vilanterol on exercise endurance in patients with chronic obstructive pulmonary disease: Two randomized, double-blind clinical trials. Ther. Adv. Respir. Dis. 8 (6), 169–181. 10.1177/1753465814559209 25452426

[B21] Naline E. Grassin Delyle S. Salvator H. Brollo M. Faisy C. Victoni T. (2018). Comparison of the *in vitro* pharmacological profiles of long-acting muscarinic antagonists in human bronchus. Pulm. Pharmacol. Ther. 49, 46–53. 10.1016/j.pupt.2018.01.003 29337266

[B22] Newman T. B. Browner W. S. Cummings S. R. Hulley S. B. (2013). “Designing studies of medical tests,” in Designing clinical Research. Editors Hulley S. B. Cummings S. R. Browner W. S. Grady D. G. Newman T. B. 4th ed. (Philadelphia, PY, USA: Lippincott Williams & Wilkins, a Wolters Kluwer business), 171–187.

[B23] Papandrinopoulou D. Tzouda V. Tsoukalas G. (2012). Lung compliance and chronic obstructive pulmonary disease. Pulm. Med. 2012, :542769. 10.1155/2012/542769 23150821 PMC3486437

[B24] Pera T. Penn R. B. (2014). Crosstalk between beta-2-adrenoceptor and muscarinic acetylcholine receptors in the airway. Curr. Opin. Pharmacol. 16, 72–81. 10.1016/j.coph.2014.03.005 24747364 PMC4096844

[B25] Pleasants R. A. Wang T. Gao J. Tang H. Donohue J. F. (2016). Inhaled umeclidinium in COPD patients: A review and meta-analysis. Drugs 76 (3), 343–361. 10.1007/s40265-015-0532-5 26755180

[B26] Restrepo R. D. (2007). Use of inhaled anticholinergic agents in obstructive airway disease. Respir. Care 52 (7), 833–851. 17594728

[B27] Salmon M. Luttmann M. A. Foley J. J. Buckley P. T. Schmidt D. B. Burman M. (2013). Pharmacological characterization of GSK573719 (umeclidinium): A novel, long-acting, inhaled antagonist of the muscarinic cholinergic receptors for treatment of pulmonary diseases. J. Pharmacol. Exp. Ther. 345 (2), 260–270. 10.1124/jpet.112.202051 23435542

[B28] Singh S. Maltais F. Tombs L. Fahy W. A. Vahdati-Bolouri M. Locantore N. (2018). Relationship between exercise endurance and static hyperinflation in a post hoc analysis of two clinical trials in patients with COPD. Int. J. Chron. Obstruct. Pulmon. Dis. 13, 203–215. 10.2147/COPD.S145285 29386889 PMC5764300

[B29] Slack R. J. Barrett V. J. Morrison V. S. Sturton R. G. Emmons A. J. Ford A. J. (2013). *In vitro* pharmacological characterization of vilanterol, a novel long-acting β2-adrenoceptor agonist with 24-hour duration of action. J. Pharmacol. Exp. Ther. 344 (1), 218–230. 10.1124/jpet.112.198481 23131596

[B30] Tal-Singer R. Cahn A. Mehta R. Preece A. Crater G. Kelleher D. (2013). Initial assessment of single and repeat doses of inhaled umeclidinium in patients with chronic obstructive pulmonary disease: Two randomised studies. Eur. J. Pharmacol. 701 (1-3), 40–48. 10.1016/j.ejphar.2012.12.019 23276660

[B31] Trivedi R. Richard N. Mehta R. Church A. (2014). Umeclidinium in patients with COPD: A randomised, placebo-controlled study. Eur. Respir. J. 43 (1), 72–81. 10.1183/09031936.00033213 23949963

[B32] Umeda A. Yamane T. Mochizuki T. Inoue Y. Tsushima K. Miyagawa K. (2019). Real-world efficacy and problems of once-daily use of inhaled steroid (fluticasone furoate) combined with long-acting beta-2 agonist (vilanterol) in Japanese patients with asthma. Cogent Med. 6, 1. 10.1080/2331205X.2019.1600632 PMC10170765 37179838

[B33] West J. B. Luks A. M. (2022b). in Airway obstruction” in west’s pulmonary pathophysiology, the essentials. Editors West J. B. Luks A. M. (Philadelphia, PY, USA: Wolters Kluwer), 70–71.

[B34] West J. B. Luks A. M. (2022a). “Maximum flows from the flow-volume curve,” in West’s pulmonary pathophysiology, the essentials. Editors West J. B. Luks A. M. (Philadelphia, PY, USA: Wolters Kluwer), 11–12.

[B35] Yoshida M. Kaneko Y. Ishimatsu A. Komori M. Iwanaga T. Inoue H. (2017). Effects of tiotropium on lung function in current smokers and never smokers with bronchial asthma. Pulm. Pharmacol. Ther. 42, 7–12. 10.1016/j.pupt.2016.11.004 27888045

[B36] Zhong N. Zheng J. Lee S. H. Lipson D. A. Du X. Wu S. (2020). Efficacy and safety of once-daily inhaled umeclidinium in asian patients with COPD: Results from a randomized, placebo-controlled study. Int. J. Chron. Obstruct. Pulmon. Dis. 15, 809–819. 10.2147/COPD.S215011 32368027 PMC7173840

